# Chemical, biochemical, and bioactivity studies on some soda lakes, Wadi El-Natrun, Egypt

**DOI:** 10.1007/s10661-024-12573-7

**Published:** 2024-04-09

**Authors:** Abeer M. A. Mahmoud, Mohamed H. H. Ali, Mohamad S. Abdelkarim, Afify D. G. Al-Afify

**Affiliations:** National Institute of Oceanography and Fisheries, Cairo, Egypt

**Keywords:** Wadi El-Natrun, Water type, Antioxidant activity, Antimicrobial activity, El-Hamra Lake

## Abstract

Wadi El-Natrun is one of the most observable geomorphological features in the North-Western Desert of Egypt; it contains several old saline and saline soda lakes. This study investigates physicochemical and biochemical characteristics and estimates the total phenolic content (TPC), total flavonoid content (TVC), and bioactivities of sediment, cyanobacteria, and brine shrimp (*Artemia salina*) in soda lakes, i.e., El-Hamra Lake 1 (H1) and El-Hamra Lake 2 (H2). These soda lakes are unique extreme ecosystems characterized by high pH (> 9.3), high alkalinity, and salinity. Some extremophilic microorganisms are hosted in this ecosystem. The results revealed that the chemical water type of studied lakes is soda-saline lakes according to the calculated percentage sequence of major cations and anions. Sodium ranked first among major cations with an abundance ratio of e% 58, while chloride came first among anions with an abundance ratio of e% 71, and bicarbonate and carbonate occupied the last rank with an abundance of 6%. The biochemical investigations showed that TPC and TVC are present in concern contents of sediment, cyanobacteria, and brine shrimp (*A. salina*) which contribute 89% of antioxidant capacity and antimicrobial activities. Thus, this study helps better understand the chemical and biochemical adaptations in soda lake ecosystems and explores natural sources with potential applications in antioxidant-rich products and environmental conservation efforts.

## Introduction

In the last decades, studying extreme ecosystems has drawn the attention of many researchers for their unique environmental characteristics that host high chemical, biotechnological, and biodiversity importance (Achour & Saadi, 2023; Hammer, 1986). Soda lakes, also known as *alkaline lakes*, have unique ecosystems characterized by extreme environmental conditions. They are found all over the world, mostly in arid and semi-arid regions (Melese & Debella, 2023), and have extreme pH levels (> 9), high conductivity (100–220 mS/cm), mostly hypersaline (> 60 ‰), high levels of sodium, carbonates, and bicarbonates (Boros, 2023; Mengistu et al., 2023). Their high salinities, alkalinity, and pH levels are often maintained by hydrological and geological factors—such as high evaporation rate, restricted precipitation, low drainage influx, and closed-type basin—which thus decrease the neutralization of alkaline substances (Boros & Kolpakova, 2018). Such ecosystems are environmentally sensitive to changes in climate and weather conditions (Jellison et al., 2004).

Salt lakes are distributed worldwide and across various African countries, particularly in the East African Rift System (EARS) where tectonic activities have led to the formation of several alkaline and saline lakes such as Lake Turkana in Kenya and Ethiopia and Lake Natron in Tanzania (Lameck et al., 2023). Other soda lakes are found in northern African countries, e.g., Wadi El-Natrun in Egypt, Chott El-Jerid in Tunisia, and Algeria (Abdelkarim et al., 2023; Achour & Saadi, 2023). Certain soda lakes are characterized by distinct colors: faint yellow, deep green, and red. Such distinctive colorations owe to the presence of pigmented microorganisms such as cyanobacterial mats, *Artemia* spp., or halophilic bacteria (Li et al., 2020). These microorganisms, including extremophilic algae and invertebrates, face great challenges to adapt and survive in such extreme conditions (Ibrahim et al., 2015; Joshi et al., 2008).

Soda lakes have notable economic impacts on local communities, where sometimes they serve as commercial sources to extract specific valuable salts—e.g., sodium chloride, sodium carbonate, and sodium sulfate—used for manufacturing purposes. The extraction of medically important ingredients from the unique extremophile microbial microorganisms potentially offers insights into the development of new pharmaceuticals or biotechnological applications (Philip & Mosha, 2012; Rampelotto, 2013), furthermore, producing novel metabolites such as enzymes, carotenoids, flavonoids, and exopolysaccharides (EPS), with antimicrobial and antioxidant activities (Achour et al., 2018). Lipase-producing and starch-degrading microorganisms were reported in Kenya’s soda lake (Hashim et al., 2004; Vargas et al., 2004). The phenolic and flavonoid compounds produced by extremophilic microorganisms have an important and essential role in environmental ecosystems due to their health impacts on different plant species and their effective antioxidant properties (Arguelles, 2022; Martínez et al., 2022). Their antioxidant activities mainly allow them to act as reducing agents and suppliers of hydrogen and oxygen (González-Ocampo et al., 2022).

Regular monitoring of the water quality of soda lakes is crucial for understanding their unique ecosystem health and dynamics and knowing the significant impacts on the balance of these ecosystems. Thus, monitoring water quality not only ensures the preservation of biodiversity but also safeguards the livelihoods of people dependent on these ecosystems. Furthermore, studying microbial diversity in these extreme environments contributes to our understanding of life’s adaptability and resilience in extreme conditions, with potential implications for astrobiology (Getenet et al. 2022).

Taher (1999) compared the Wadi El-Natrun soda lake ecosystem with that of the Dead Sea and the Great Salt Lake (Utah). As concluded, despite that the three ecosystems have similar salinities, Wadi El-Natrun appears unique among saline soda lakes—which are characterized by alkaline brines poor in Ca^+2^ and Mg^+2^. The diluted HCO_3_^−^-CO_3_^−2^ spring water evolves into an alkaline Na-SO_4_-Cl-rich brine and sulfate is lost as H_2_S and/or metal sulfide, while the increased C1^−^ enhances metal solubility due to the soluble chloro-complexes formation. These are the main characteristics of Wadi El-Natrun Soda Lakes. In addition, the mass development of cyanobacteria, halophilic phototrophic sulfur bacteria, sulfate-reducing bacteria, and algae is extremely characteristic.

Hence, this study mainly aims to understand the unique ecosystems, elucidate microbial diversity, investigate extremophile adaptations, and uncover potential applications in biotechnology and environmental remediation of El-Hamra Soda Lakes in the Wadi El-Natrun depression. This shall also contribute to the conservation and sustainable management of this distinctive extreme ecosystem.

## Materials and methods

### Description of sampling site

**Fig. 1:**
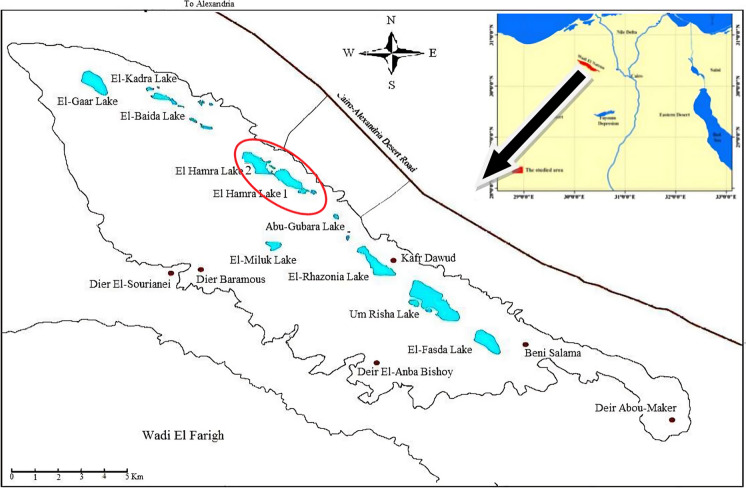
Maps showing the location of Wadi El-Natrun Lakes and El-Hamra Lakes: H1 and H2 (red circle)

Wadi El-Natrun is an elongated depression at 23 m below sea level in the western desert of Egypt, situated between latitudes 30°17′N to 30°19′N and longitudes 30°10′E to 30°25′E (Fig. 1). Extending approximately 60 km from north to south, this depression is renowned for its historical and ecological significance and characterized by a series of hypersaline lakes, including Um Risha, Ruzita, El-Khadra, Abou Jiyad, and El-Hamra. Some lakes completely evaporated with precipitated minerals on the overlying sediment, whereas others are still filled with water mainly regenerated from the underground regime (Abdelkarim et al., 2023). The two hypersaline lakes, i.e., El-Hamra Lake 1 (H1) and El-Hamra Lake 2 (H2), are in the middle of Wadi El-Natrun Lakes. Their native name *El-Hamra* (i.e., red color) is due to the flourishing of brine shrimp: *A*. *salina.*

### Sampling collections

In this study, the different samples, including the subsurface water, sediment, cyanobacterial mats, and *A. salina*, were collected from H1 and H2 from autumn 2021 to summer 2022. Four sites were sampled a few meters from the shoreline of each lake. *A. salina* exclusively was collected during the low-temperature period from late autumn to late winter, while cyanobacterial mats were not always available at all sites in both lakes. By the end of the sampling program, about 24 water samples, 24 sediment samples, 18 cyanobacterial mats samples, and 12 *A. salina* samples were collected. Due to the difference in the number of collected samples, the results of a specific material in one lake at a specific sampling date were pooled together. Water samples were collected by using a 2.5-L Ruttner water sampler. The samples were immediately kept in dry labeled polyethylene bottles for chemical analysis. Sediment samples were collected using the Ekman grab and then kept in clean and dry labeled polyethylene bags. Cyanobacteria samples were collected by hand and kept in sterile, clean, and dry polyethylene bags. *A. salina* was collected using a plankton net of 50 µm mesh size.

### Chemical analysis

Water temperatures, electrical conductivity (mS/cm), and pH were determined in the field by using a Multiparameter HANNA device: HI9829. The methods of the American Public Health Association (APHA, 2017) were applied to evaluate different chemical parameters.

### Biochemical analysis

Homogenized wet samples of sediment, cyanobacterial mats, and *A. salina* were used to estimate the total protein, total lipid, carbohydrates, *ß*-carotene, and phycocyanin. Different biochemical contents, except for phycocyanin, were determined against the corresponding standard curve, while the sample concentration was estimated from the following equations:

1 $$y={b}_{0}+{b}_{1}x$$

where *y* is the absorbance and *x* is the biochemical content concentration. The constants: *b*_0_ and *b*_1_ are the calibration curve’s expected *y*-intercept and its expected slope, respectively.

Therefore:

2 $$x=\frac{(y-{b}_{0})}{{b}_{1}}$$

#### Total protein

The Biuret method (David & Hazel, 1993) was used for the total protein estimation. Copper in an alkaline medium reacted with the peptide bond giving a blue color. An amount of 10 mg of sample and the standard bovine albumin were grinded in 10 ml saline solution (0.15 M NaCl, *w/v*) and then centrifuged, while 5 ml of the Biuret reagent was added to 0.1 ml of the saline extract and bovine albumin. Distilled water was used as blank. Samples with reagents were kept at room temperature for 30 min, and the blue color was measured spectrophotometrically at 550 nm. The standard curves of bovine albumin concentrations and corresponding absorbances were plotted, and the regression coefficient was 0.991. Total protein contents were determined according to Eqs. 1 and 2.

#### Total lipid

Total lipid was determined by using the method of Chabrol and Castellano (1961). Total lipid contents were determined by the sulfo-phospho-vanillin (SPV) procedure. An amount of 10 mg of samples and cholesterol was transferred to dried glass tubes, and 5 ml of concentrated sulfuric acid was added and boiled in a water bath for 10 min. After complete digestion, 3 ml of the phosphor-vanilline reagent was added to 0.2 ml samples in test tubes and left in the dark for 45 min. The absorbance of the characteristic pale pink color was measured spectrophotometrically at 525 nm. The standard curve of cholesterol concentrations showed a regression coefficient of 0.987, and the total lipid contents were estimated according to Eqs. 1 and 2.

#### Total carbohydrates

Carbohydrate content was hydrolyzed according to the method modified by Myklestad and Haug (1972) and measured by using the phenol–sulfuric acid method (Dubois et al., 1956). An amount of 2 ml of sulfuric acid (80%) was added to 10 mg of samples and the standard glucose in dried glass test tubes and kept at 20 °C. After 20 h, the samples were completed to 5 ml by using distilled water, and 1 ml of phenol was added to 0.5 ml of the samples and the standard. After vigorous shaking, 5 ml of concentrated sulfuric acid was added. After 10 min, the samples were shaken again and kept for 20 min at 25 °C. The absorbance of the yellow-orange color was measured spectrophotometrically at 480 nm. The regression coefficient of the standard glucose curve was 0.993, and the total lipid contents were estimated according to Eqs. 1 and 2.

#### ß-carotene

*ß*-carotene was measured by using the method described by Davies (1976) against the *ß*-carotene standard. About 50 ml of methylene chloride was added to a specific weight of the samples and the standard and left overnight at − 4 °C in the refrigerator. Samples were dried at 35 ± 2 °C in Memmert Oven UNB 100 under forced-air circulation. After complete dryness, the residue was dissolved in 10 ml of petroleum ether and measured spectrophotometrically at 451 nm. *ß*-carotene contents in different samples were determined according to Eqs. 1 and 2 from the resulting standard curve. The regression standard curve showed a highly strong correlation coefficient (*R*^*2*^ = 0.991).

#### Phycocyanin contents

Phycocyanin contents were determined spectrophotometrically as described by Patel et al. (2005) against the phycocyanin standard. Dry weight (0.1 g) of samples and phycocyanin standard were suspended in 10 ml of 0.15 M phosphate buffer (pH = 7.0) and left at 4 °C for 24 h. The samples were centrifugated at 13,000 rpm for 15 min. The blue color intensity of the supernatant of samples and phycocyanin standard was measured at wavelengths of 615 and 652 nm (Eq. 3).

3 $$\mathrm{Phycocyanin}\;(mg/g)=\frac{{OD}_{615}-{0.474OD}_{652}}{5.34}$$

where OD615 is the optical density of the sample at 615 nm and OD652 is the optical density of the sample at 652 nm.

### Estimation of total flavonoid contents (TFC), total phenolic contents (TPC), and assay of free radical scavenging activity by the DPPH method

#### Samples preparation

Sediment, cyanobacterial mats, and *A. salina* samples were dried under forced-air circulation at 35 ± 2 °C in a drying oven until weights stabilized. Dry biomass (15 gm) of each component was sonicated for 30 min in 100 ml of ethyl acetate, methanol, acetone, chloroform, and n-hexane and then left overnight. All extracts were filtered on a Whatman filter paper (No. 1) and then evaporated under vacuum till dryness. After complete dryness, the extracts were weighed and dissolved in dimethyl sulfoxide (DMSO) at a concentration of 5 mg/ml. Extracts were stored in glass vials in the dark at − 20 °C.

#### Total flavonoid contents

The total flavonoid content in methanolic extract was estimated by the aluminum chloride colorimetric method and measured spectrophotometrically at 415 nm by using a double-beam UV–Vis spectrophotometer. The amount of flavonoid in the mentioned samples was estimated according to a linear regression equation against the quercetin calibration curve and expressed as mg/g of quercetin equivalent (QE) of dry extract (1 g) (Bag et al., 2015). The regression line of the QE curve showed a strong correlation coefficient (*R*^*2*^ = 0.991), and the flavonoid contents were determined according to Eqs. 1 and 2.

#### Determination of total phenolic contents (TPC)

The total phenolic content (TPC) of the methanolic extract was estimated according to the Folin-Ciocalteu reagent by using the double-beam UV–Vis spectrophotometer. Gallic acid was used as a standard, while the absorbance of the resulting blue color was measured at 765 nm. TPCs were calculated by the linear regression equation obtained from the standard plot of Gallic acid and expressed as mg/g gallic acid equivalent (GAE) of dry extract (1 g) (Bag et al., 2015). The performed standard curve was used to quantify polyphenols in the mentioned samples according to Eqs. 1 and 2. The regression of the standard curve showed a highly strong association (*R*^*2*^ = 0.9877).

#### Assay of free radical scavenging activity by DPPH method

The free radical scavenging activities of the methanolic extracts of sediment, cyanobacterial mats, and *A. salina* were evaluated by the DPPH (1, 1-diphenyl-2-picrilhydrazyl) radical scavenging method. Ascorbic acid was used as a positive reference standard. Various crude methanolic extracts, 4, 6, 8, 10, 20, 30, 40, and 50 mg/mL of different samples, and five concentrations, 0.2, 0.4, 0.6, 0.8, and 1 mM of ascorbic acid standard solution, were taken in separate test tubes. Two milliliters of 0.5 mmol/L DPPH radical solution, dissolved in methanol, was added to each test tube. The solution was rapidly mixed and allowed to stand in the dark at 37 °C for 30 min. The blank was prepared in a similar way without extract or ascorbic acid. The absorbance of each solution was measured at 517 nm by using a UV–Vis spectrophotometer (Bag et al., 2015). The percentage of radical scavenging activities of tested extracts and ascorbic acid were calculated according to the following equation (Eq. 4):

4 $$\mathrm{DPPH\;Scavenged\;}\left(\mathrm{\%}\right)=\frac{Ac-As}{Ac}\;x\;100$$

where Ac is the absorbance of control (methanol + DPPH) at 517 nm and As is the absorbance of sample.

#### In vitro*, antimicrobial, and antifungal assays*

##### Tested microorganisms

Two gram-negative bacterial strains, Salmonella typhi (S. typhi) and Pseudomonas aeruginosa (P. aeruginosa), two gram-positive bacterial strains, Staphylococcus aureus (S. aureus) and Bacillus cereus (B. cereus), and the fungal Candida albicans (C. albicans) were used for antimicrobial assays. The parent cultures were obtained from the Hydrobiology Laboratory at the National Institute of Oceanography and Fisheries (NIOF), and the subcultures were maintained once every 15 days.

##### Antimicrobial assays

The antimicrobial activities of sediment, cyanobacterial mats, and A. salina in ethyl acetate, methanol, acetone, chloroform, and n-hexane extracts were screened by using Mueller Hinton Agar (MHA) (Oxoid Ltd. Basing Stoke, Hampshire, England). Molten MHA media (15 ml) were poured into sterile petri plates, solidified for 5 min, and then a standardized bacterial inoculum was uniformly spread on the MHA. Wells of 5 mm in diameter were made through the agar surface by using a sterile metallic cylinder. An amount of 50 μl of DMSO-containing extract was injected into the well. All tests were done in triplicate. The plates were incubated at 37 °C for 24 h, and the inhibition zones around the wells were measured in ml. A well containing only DMSO was used as a negative control.

### Data analyses

Pearson correlation coefficient matrix was used to relate the physico-chemical characteristics to biochemical contents; protein, carbohydrate, lipid, ß-carotene, phycocyanin (*p* < 0.05). To compare seasons and different sites, a one-way ANOVA was performed. Pearson correlation coefficient and a one-way ANOVA were performed by using XLSTAT 2016.

## Results and discussion

### Cyanobacterial mat species composition

Cyanobacterial mat is a thin layer of 1–2 mm existing throughout the year along the lakes’ margins. Filamentous cyanobacterial forms are the most present. *Lyngbya* spp. (particularly *L. scottii*), *Microcoleus chthonoplastes* and *M. tenerrimus, Arthrospira major*, *Schizothrix funiculus*, and *Phormidium* spp*.* (particularly *P. retzii*) are more abundant. The coccoid *Gloeocapsa* spp. (particularly *G. magna* and *G. compacta*) are the most present coccoid forms, in addition to *Synechococcus aeruginosus* and *Synechocystis* sp. Cyanobacteria are the most alkaliphilic microbes that frequently dominate soda lakes and microbial mats (Dadheech et al., 2013; Kalwasinska et al., 2017). Taher and Abdel-Motelib, 2015) recorded the filamentous *Microcoleus chthonoplastes*, *Spirulina* sp., *Lyngbya* sp., and the coccoid *Synechococcus* sp. in the mudflat of the alkaline hypersaline lakes of Wadi El-Natrun. Abd El-Karim and Goher (2016) found that *Lyngbya* spp., the coccoid *Syncechococcus* spp., and *Synechocystis* spp. were highly present in the cyanobacterial mats in some western-desert hypersaline lakes, Egypt.

### Physico-chemical characteristics

Table 1 shows the measured physicochemical characteristics of water of the Wadi El-Natrun Soda Lakes during the study period. The water temperature varied between 19.3–33.2 °C and 19.2–34.1 °C in H1 and H2, respectively. There was a remarkable increase in water temperature during summer than the corresponding values during winter (Table 1). TDS contents ranged between 122.3–265.8 g/L and 120.2–161.7 g/L for H1 and H2, respectively, during different sampling periods, while EC varied between 137.5–285.0 mS/cm and 127.7–206.8 mS/cm, respectively. There was a remarkable increase in TDS and EC values in H1 than H2 owing to the inflow of agricultural effluents from the neighboring agricultural lands surrounding H2 leading to some water dilution. Both H1 and H2 recorded high pH values ranging between 9.15–9.26 and 9.13–9.26, respectively. A notable increase in pH values during winter (avr*.* 9.22) compared to the summer season (*avr.* 9.14). On the other hand, the elevation of pH values can be explained based on silicate mineral hydrolysis that contains high HCO_3_^−^ and CO_3_^2−^ concentrations (Deocampo & Jones, 2013; Yona et al., 2023).

**Table 1:** Range, mean and standard deviation (SD) of some physico-chemical characteristics of El Hamra 1 (H1) and El Hamra 2 (H2) soda lakes

Site parameters		Autumn				Winter				Spring				Summer			
		H1		H2		H1		H2		H1		H2		H1		H2	
		Range	Mean ± SD	Range	Mean ± SD	Range	Mean ± SD	Range	Mean ± SD	Range	Mean ± SD	Range	Mean ± SD	Range	Mean ± SD	Range	Mean ± SD
Hydrographic parameters	W ^0^C	22.1–26.5	24.6 ± 1.9	22.2–26.5	24.6 ± 1.6	19.3–19.4	19.4 ± 0.07	19.2–19.3	19.3 ± 0.07	26.6–26.9	26.8 ± 0.2	26.4–26.7	26.5 ± 0.2	32.7–33.2	32.9 ± 0.3	33.1–34.1	33.6 ± 0.7
	TS g/l	124.5–268.8	195.4 ± 50.3	124.5–163.5	140.7 ± 10.2	181.0–183.6	182.3 ± 1.8	123.1–125.4	124.2 ± 1.6	192.2–192.8	192.5 ± 0.4	123.0–124.1	123.6 ± 0.7	195.8–196.5	196.2 ± 0.4	151.2–156.4	153.8 ± 3.7
	TDS g/l	122.3–265.8	193.3 ± 50.2	122.6–161.7	138.8 ± 10.2	178.3–181.2	179.7 ± 2.0	121.8–123.6	122.7 ± 1.3	189.8–190.2	190.0 ± 0.2	120.2–120.8	120.5 ± 0.4	191.8–192.4	192.1 ± 0.4	145.0–146.4	145.7 ± 1.0
	EC mS/cm	137.5–285.0	223.0 ± 56.0	127.7–198.1	178.7 ± 26.0	254.3–261.3	257.8 ± 4.9	169.6–181.2	175.4 ± 8.2	265.8–266.3	266.0 ± 0.3	171.5–175.6	173.6 ± 2.8	268.0–268.4	268.2 ± 0.3	203.9–206.8	205.3 ± 2.0
	pH	9.15–9.26	9.21 ± 0.04	9.16–9.26	9.22 ± 0.04	9.21–9.23	9.22 ± 0.01	9.17–9.19	9.18 ± 0.01	9.19–9.22	9.20 ± 0.02	9.13–9.15	9.14 ± 0.02	9.22–9.22	9.22 ± 0.00	9.14–9.16	9.15 ± 0.01
Oxygen studies	DO mg/l	0.92–8.08	4.46 ± 2.71	3.40–11.12	5.91 ± 2.79	3.36–7.52	5.44 ± 2.94	4.80–6.12	5.46 ± 0.93	4.28–4.56	4.42 ± 0.2	4.22–4.43	4.32 ± 0.15	3.30–3.40	3.35 ± 0.07	3.25–3.30	3.28 ± 0.04
	BOD mg/l	0.48–3.36	1.58 ± 1.18	2.68–6.63	4.27 ± 1.42	1.72–6.20	3.96 ± 3.17	2.36–4.92	3.64 ± 1.81	3.85–3.90	3.88 ± 0.04	3.83–3.85	3.84 ± 0.02	1.90–2.00	1.95 ± 0.07	3.70–3.75	3.73 ± 0.04
	COD mg/l	16.0–31.0	22.1 ± 5.3	7.0–22.1	17.1 ± 6.1	10.3–20.8	15.6 ± 7.4	11.0–20.1	16.00 ± 5.8	16.3–16.4	16.3 ± 0.1	22.1–22.5	22.2 ± 0.3	23.8–24.0	23.9 ± 0.1	23.4–23.5	23.4 ± 0.1
Major anions	CO_3_^−2^ g/l	2.75–4.14	3.66 ± 1.23	2.50–3.75	2.79 ± 1.08	2.36–2.73	2.54 ± 0.26	2.50–2.85	2.68 ± 0.25	2.24–2.25	2.24 ± 0.01	1.91–1.98	1.94 ± 0.05	2.30–2.45	2.39 ± 0.01	1.05–1.10	1.08 ± 0.04
	HCO_3_^−^ g/l	3.75–11.00	6.66 ± 3.79	3.94–6.75	5.32 ± 2.26	5.65–5.88	5.76 ± 0.16	3.55–3.84	3.69 ± 0.20	3.20–3.31	3.30 ± 0.02	3.04–3.10	3.07 ± 0.05	4.24–4.26	4.25 ± 0.01	1.68–1.73	1.70 ± 0.04
	Cl^−^ g/l	76.1–78.5	75.7 ± 4.0	51.1–56.7	54.1 ± 4.3	66.8–73.5	70.3 ± 4.7	48.5–50.1	49.4 ± 1.1	65.6–73.8	70.7 ± 0.15	47.4–48.6	47.5 ± 0.18	74.4–77.4	74.93 ± 0.7	49.6–54.4	52.5 ± 1.2
	SO_4_^−2^ g/l	22.6–29.7	25.3 ± 10.2	19.2–29.4	23.9 ± 7.3	20.3–33.1	30.7 ± 9.0	20.8–24.9	22.8 ± 2.9	28.6–28.9	28.7 ± 0.2	21.6–23.8	22.7 ± 0.1	28.1–28.2	28.1 ± 0.1	24.4–24.6	24.5 ± 0.1
Major cations	Ca^+2^ g/l	1.6–2.8	2.1 ± 0.7	1.7–3.0	2.2 ± 0.7	2.6–2.8	2.7 ± 0.2	1.6–1.8	1.7 ± 0.1	2.4–2.5	2.4 ± 0.1	1.3–1.4	1.39 ± 0.01	2.1–2.1	2.1 ± 0.03	2.2–2.88	2.5 ± 0.1
	Mg^+2^ g/l	18.1–22.1	20.6 ± 3.7	15.7–18.3	16.6 ± 9.5	20.1–23.4	22.2 ± 0.01	15.5–18.2	17.0 ± 0.01	20.8–21.3	20.3 ± 1.4	17.1–18.9	18.0 ± 1.3	22.8–24.4	23.7 ± 1.1	18.4–20.3	19.9 ± 0.6
	Na^+^ g/l	33.0–49.9	40.2 ± 2.6	23.6–30.7	27.4 ± 2.8	39.3–39.3	39.3 ± 0.1	26.8–26.9	26.8 ± 0.08	41.7–42.2	41.9 ± 0.3	25.7–27.3	26.0 ± 0.4	40.6–42.7	41.7 ± 0.02	27.9–29.1	28.0 ± 0.1
	K^+^ g/l	4.0–6.0	5.0 ± 0.9	3.0–5.0	4.02 ± 0.6	5.03–5.04	5.03 ± 0.01	4.02–4.14	4.08 ± 0.08	5.29–5.46	5.37 ± 0.12	5.22–5.87	5.54 ± 0.46	6.31–6.34	6.32 ± 0.02	5.58–5.66	5.62 ± 0.06
Nutrient salts	NO_2_^−^ µg/l	19.4–45.1	30.3 ± 11.0	8.8–60.4	24.7 ± 20.3	10.9–25.5	18.2 ± 10.3	7.8–71.1	39.4 ± 44.8	19.1–20.1	19.6 ± 0.7	33.0–33.5	33.2 ± 0.6	100.7–110.6	109.6 ± 1.3	91.22–95.41	93.31 ± 2.97
	NO_3_^−^ µg/l	10.0–57.3	25.0 ± 19.3	3.2–79.8	29.2 ± 21.6	8.0–9.2	8.6 ± 0.9	9.2–14.3	11.7 ± 3.6	2.2–2.4	2.3 ± 0.1	2.0–2.1	2.0 ± 0.1	11.4–12.2	11.8 ± 0.5	4.60–5.42	5.01 ± 0.58
	NH_4_ µg/l	227–1273	697.3 ± 354	355–1876	1066 ± 654	469–1602	1035 ± 801	1081–1091	1085. ± 0.3	585–594	589 ± 6	587–589	588 ± 1	2096–2131	2113 ± 24	1784–1792	1788 ± 5.8
	PO_4_^−3^ mg/l	1.10–4.35	2.38 ± 1.34	1.31–2.01	1.64 ± 0.27	1.20–1.37	1.29 ± 0.12	1.29–1.58	1.43 ± 0.20	1.05–1.06	1.06 ± 0.01	1.08–1.09	1.09 ± 0.01	0.95–0.97	0.96 ± 0.01	0.49–0.51	0.50 ± 0.01
	TP mg/l	1.86–5.58	3.34 ± 1.42	1.95–3.62	2.73 ± 0.67	1.94–2.01	1.98 ± 0.05	2.02–2.38	2.2 ± 0.26	1.67–1.68	1.68 ± 0.01	1.66–1.67	1.67 ± 0.01	1.64–1.72	1.67 ± 0.05	0.91–0.94	0.93 ± 0.02
	SiO_2_^−^ mg/l	5.47–26.38	9.85 ± 6.03	6.76–13.09	10.31 ± 2.26	7.20–11.46	9.33 ± 3.01	6.42–8.87	7.65 ± 1.73	6.68–6.87	6.77 ± 0.14	9.90–9.97	9.93 ± 0.05	22.09–23.14	22.61 ± 0.74	24.42–24.63	24.53 ± 0.15

The values of DO, BOD, and COD in H1 and H2 during different sampling times were 0.9–8.1 mg/L and 3.4–11.1 mg/L; 0.5–6.2 mg/L and 2.4–6.6 mg/L; and 10.3–31.0 and 7.0–23.5 mg/L, respectively (Table 1). A relative increase in DO values was recorded during the autumn season (*avr.* 5.91 mg/L) in H2, while the lowest DO mean value was recorded in H1 during summer (*avr.* 3.28 mg/L). This is due to increasing salinity in H1 and temperature elevation during summer (Abdelkarim et al., 2023). The average values of both BOD and COD at H2 (*avr.* 3.97–19.83 mg/L) were comparatively higher than H1 (*avr.* 2.93–18.96 mg/L). A high positive significant correlation was recorded between DO and BOD (*r* = 0.84; *p* < 0.05), while a moderate significant correlation between DO and COD (*r* = 0.48; *p* < 0.05) (Table 4) was observed.

Studying the concentration and distribution of major cations, Na^+^, K^+^, Ca^+2^, and Mg^+2^, and major anions, CO_3_^−2^, HCO_3_^−^, Cl^−^, and SO_4_^−2^, was extremely essential to determining the type of water and the chemical interactions between these ions with the rocks and underlying sediment (Subramani et al., 2010). The disparity in salinities between H1 and H2 was manifested in distinct concentrations of major cations and anions, delineating a contrast in their chemical compositions. H1 is characterized by higher salinity levels and shows higher concentrations of both major cations and anions than H2. The obtained results showed that the Na^+^ ion was the most abundant dissolved cation; it reached 58.3% and 53.0% of the total dissolved cations in meq/L with annual averages of 41.08 g/L and 27.11 g/L in H1 and H2, respectively (Table 1 and 3). Magnesium (Mg^+2^) was found in the second rank of the dominant cations with 35.2% and 39.3% meq/L with annual averages of 21.77 g/L and 17.71 g/L in both H1 and H2. It is notable that Na^+^ and Mg^+2^ contents represent more than 93% of total dissolved cations. Potassium (K^+^) contents contribute about 4.6% and 5.5% with annual average content of 5.44 g/L and 4.81 g/L in H1 and H2. Ca^+2^ was a less dominant cation among the total dissolved cations reaching 1.8% and 2.1% with annual average content of 2.25 g/L and 1.91 g/L in both H1 and H2 (Table 1 and 3).

Furthermore, it is notable that the concentration of monovalent cations (Na^+^ and K^+^) exceeds the concentration of divalent cations (Ca^+2^ and Mg^+2^) in both studied lakes, indicating the dominancy of alkali metals more than earth alkali metals due to the ion exchange process (Li et al., 2018). The dominancy of major cations was found in the order of Na^+^  > Mg^2+^  > K^+^  > Ca^2+^.

Chloride ion was the most dominant dissolved anion in H1 and H2; it contributed 72.9% and 69.6% with annual averages of 72.90 g/L and 50.90 g/L, respectively. Sulfate was the next abundant dissolved anion contributing 20.9% and 23.7% with annual averages of 28.25 and 23.52 g/L. The contribution ratio of HCO_3_^−^ + CO_3_^2−^ in H1 and H2 was lower than other anions. CO_3_^−2^ contributed with 2.71 and 2.19 g/L (i.e., 3.2–3.5%), while HCO_3_^−^ came last abundant anions which contributed with annual averages of 5.24 and 3.95 g/L (i.e., 3.0–3.1% of the total anions). The sequence dominance of total anions in El-Hamra Lakes was found in the order of Cl^−^ > SO_4_^2−^  > CO_3_^2−^  > HCO_3_^−^ (Table 1 and 3). There are high positive significant correlations between major dissolved ions with pH values (*r* = 0.72, 0.77, 0.63, 0.72, and 0.83) at *p* < 0.05 with CO_3_^−2^, HCO_3_^−^, Cl^−^, SO_4_^−2^, and Ca^+2^, respectively (Table 4). Table 2 shows a comparative distribution of major ions: Mg^2+^, Ca^2+^, Na^+^, K^+^, CO_3_^−^, HCO_3_^−^, SO_4_^2−^, and Cl^−^, and pH in different lakes: soda, soda saline, and saline lakes from Brazil, Ethiopia, Egypt, Tanzania, Kenya, and Uganda.

**Table 2:** Comparative of pH, major anions, and major cations of El-Hamra soda lakes with other global soda lakes

Lake parameter	El Hamra (Egypt)	El Hamra (Egypt)	Nyamunuka (Uganda)	Nhecolândia (Brazil)	Bogoria (Kenya)	Nasikie Engida (Kenya)	Nakuru (Kenya)	Sonachi (Kenya)	Bishoftu (Ethiopia)	Manyara (Ethiopia)	Chitu. (Ethiopia)	Eyasi (Tanzania)	Natron (Tanzania)
pH	9.19	9.51	9.27	9.7	10.0	9.59	9.8	9.5	10.1	10.3	10.1	9.55	9.63
CO_3_^−2^ g/l	2.7	5.50	1.226	*	11.36	33.20	6.10	39,85	1.52	0.22	0.26	0.16	4.11
HCO_3_^−^ g/l	5.2	2.95	19.27	*	30.29	11.60	9.63	20,56	24.48	3.27	3.89	2.34	131.17
Cl^−^ g/l	72.8	60.92	4.21	0.19	6.58	137,20	5.25	30,05	12.33	0.78	0.48	2.24	36.51
SO_4_^−2^ g/l	28.3	84.90	0.48	0.01 l	0.613	56,30	0.10	7.52	0.188	0.34	0.02	0.28	2.61
Ca^+2^ g/l	2.3	0.02	1.0	0.007	0.0	1.40	4.5	2.0	0.0	0.07	0.014	0.04	3.0
Mg^+2^ g/l	21.8	0.18	0.0	0.001	3.0	0.0	12.0	0.0	0.0	0.70	0.0	0.010	3.0
Na^+^ g/l	41.1	84.66	9.9	0.43	27.52	136.00	12.85	47,62	20.50	0.36	1.62	2.77	67.89
K^+^ g/l	5.5	0.56	0.37	0.14	0.73	6.80	2.28	144	1.20	0.06	0.17	0.0	0.65
References	This study	Hamed et al. (2007)	Gizaw (1996)	Guerreiro et al. (2019)	Jirsa et al. (2013)	Arad and Morton (1969)	Getenet et al. (2022)	Renaut et al. (2021)	Ogato et al. (2014)	Wood and Talling (1988)	Ogato et al. (2015)	Deocampo (2005)	Fritz et al. (1987)

*Not available

NO_2_^−^-N values varied in the range of 10.9–110.6 µg/L and 7.80–95.4 µg/L with annual averages of 43.9 µg/L and 50.15 µg/L in H1 and H2, respectively. NO_3_^−^-N values showed an observable decrease than NO_2_^−^ in both lakes. Their values fluctuated between 2.2–45.1 µg/L and 2.0–79.8 µg/L with annual averages of 14.09 µg/L and 15.08 µg/L. NH_4_^+^-N values showed higher values than NO_2_ and NO_3_. Their values ranged between 227–2131 µg/L and 355–1876 µg/L with annual averages of 1122 µg/L and 1144 µg/L. The main reason for elevated ammonium values is the agricultural wastewater inflow to the lakes from neighboring agricultural lands. Orthophosphate (PO_4_^−3^) and total phosphorus (TP) exhibited similar distribution patterns at sampling sites showing high values. PO_4_^−3^ fluctuated between 1.0–4.4 mg/L and 0.5–2.0 mg/L with annual averages of 1.51 mg/L and 1.17 mg/L in H1 and H2, respectively, while TP values fluctuated between 1.6–5.6 mg/L and 0.9–3.6 mg/L with annual averages of 2.26 mg/L and 1.89 mg/L, respectively.

### Chemical water type

The metamorphic coefficients: K1 and K2 were calculated from Eqs. 4 and 5 (Table 3). K1 (i.e., soda index) showed the relationship between the concentration of CO_3_^−2^ + HCO_3_^−1^ with the concentration of Ca^+2^ + Mg^+2^, while K2 showed the relationship between the concentration of CO_3_^−2^ + HCO_3_^−1^ + SO_4_^−2^ with the concentration of Ca^+2^ + Mg^+2^. These indices are widely used to determine the water chemical type according to their values. When K1 and K2 values are < 1, the water type is chloride. If K2 value is close to 1 (K2 ≤ 1), the water is of the sulfate type. The values of calculated K1 and K2 in El-Hamra Lakes were 0.16 and 0.68, respectively (Table 3), and therefore the major chemical water type is chloride-sulfate. Boros and Kolpakova (2018) classified the lakes according to the dominancy of Na^+^ and the summation of HCO_3_^−^  + CO_3_^2−^ as follows:

**Table 3:** Average percentage (e%) of major cations and anions and the type of water in El-Hamra Lakes

	Avr. major anions (e%)				Avr. major cations (e%)				Soda index		Water type
	CO_3_^−2^	HCO_3_^−^	SO_4_^−2^	Cl^−^	Ca^+2^	Mg^+2^	Na^+^	K^+^	K_1_ (Eq. 5)	K_2_ (Eq. 6)	
H1	3.2%	3.0%	20.9%	72.9%	1.8%	35.2%	58.4%	4.6%	0.16	0.68	Soda saline lake
H2	3.5%	3.1%	23.7%	69.6%	2.1%	39.3%	53.0%	5.5%	0.15	0.68	Soda saline lake

- Soda-type class, where Na^+^ and HCO_3_^−^  + CO_3_^−2^ are the first dominant e% > 25.
- Soda-saline class, where Na^+^ is the first dominant e% > 25, while HCO_3_^−^ + CO_3_^−2^ is less than Cl^−^ or SO_4_^2−^ e%.

As revealed in the results, Na^+^ ranked first in cations abundance (58%), and the summation of HCO_3_^−^  + CO_3_^2−^ (≈ 6%) was less than the abundance of Cl^−^ (≈ 71%) and SO_4_^−2^ (≈ 22%). El-Hamra Lakes were accordingly classified as a *soda-saline* chemical type (Boros & Kolpakova, 2018).

5 $$K1= \frac{{CO}_{3}^{-2}+{HCO}_{3}^{-}}{{Ca}^{+2}+ {Mg}^{+2}}$$

6 $$K2= \frac{{CO}_{3}^{-2}+{HCO}_{3}^{-}+ {SO}_{4}^{-2}}{{Ca}^{+2}+ {Mg}^{+2}}$$

**Fig. 2:**
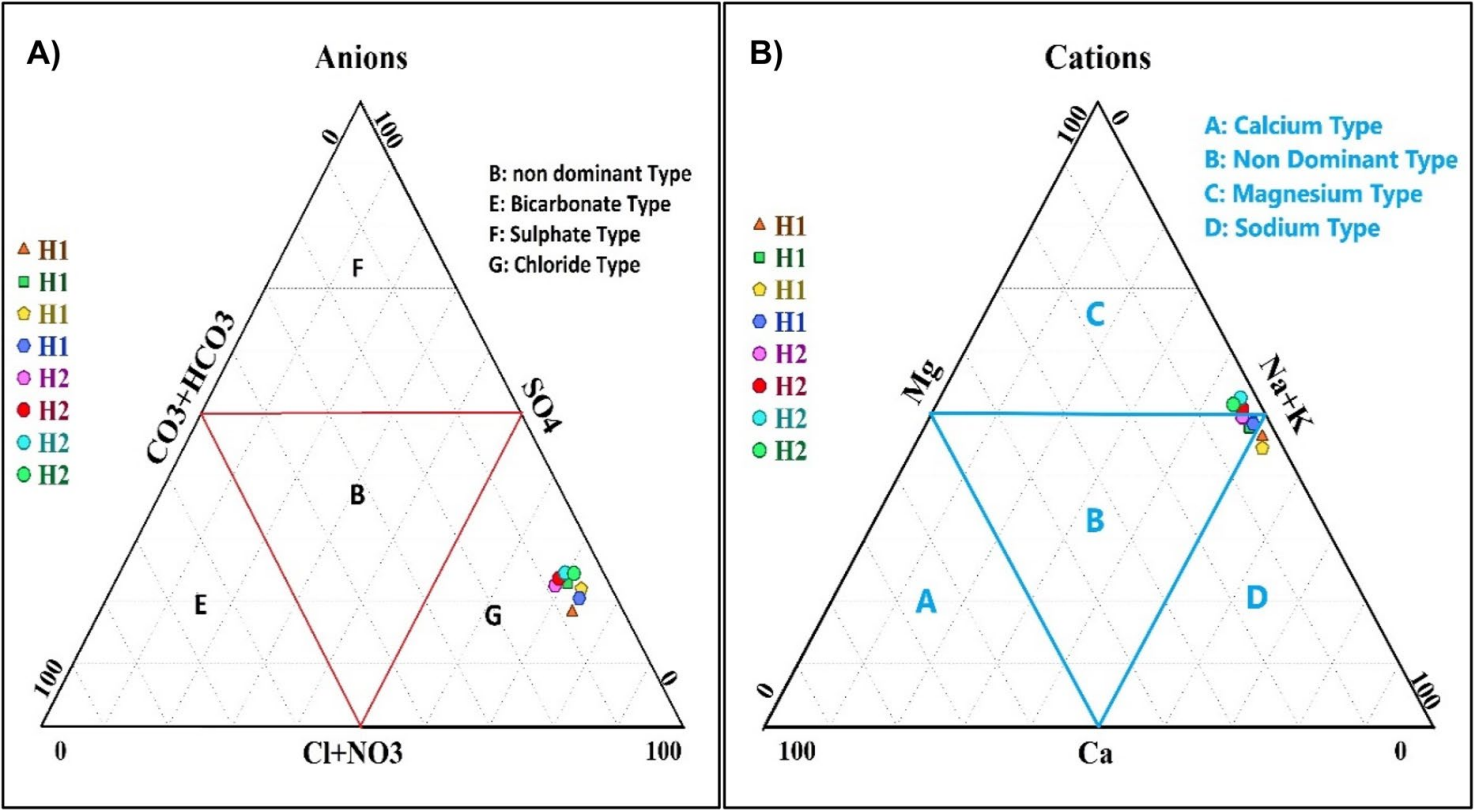
Ternary diagrams of the equivalent percentage contribution of **A** major anions and **B** major cations to the total dissolved ions

According to the ionic composition of El-Hamra Lakes, the ternary diagrams (Fig. 2A and B) reveal that the cation composition is dominated by Na^+^ and Mg^+2^, contributing more than 93% of the equivalent percentage. Therefore, the two types of water are sodium type and magnesium type. In terms of dissolved anions, Cl^−^ came first with an equivalent percentage > 71%. Thus, the water of El-Hamra Lakes is of the chloride type (Arnuk et al., 2023; Boros et al., 2017).

### The biochemical contents of sediment

The maximum protein and lipid contents were detected in H2 during summer (152.38 and 4.24 mg/100 g wet wt., respectively). The elevation of water temperature enhanced the metabolic rate and induced an increase in some biochemical compounds especially protein and lipid contents because of the obvious thin pink bacterial film flourishing or the sediment can act as a trap for the de-attached cyanobacterial fragments and brine shrimp cyst (Bibi et al., 2021). Furthermore, strong positive significant correlations between protein and lipid with temperature were observed (*r* = 0.0.9 and 0.6 at *p* > 0.05) (Table 4).

**Table 4:**
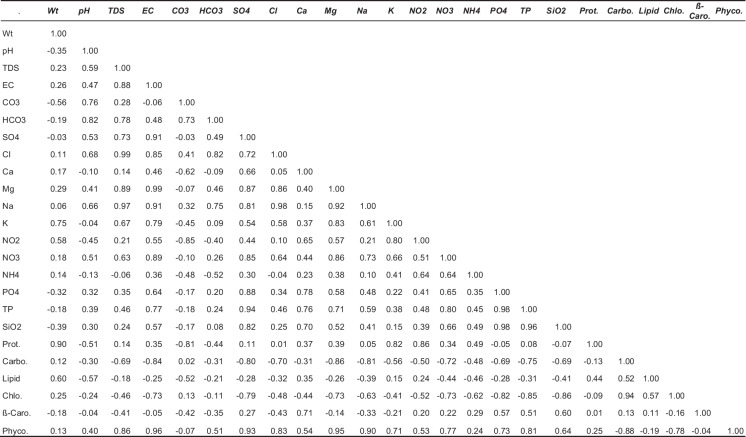
Correlation matrix between physicochemical characteristics with biochemical contents of the studied area, *p* < 0.05

**Fig. 3:**
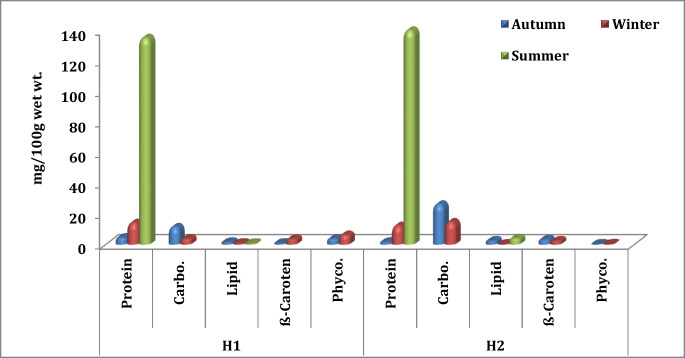
Biochemical contents of El-Hamra Lake sediment

The maximum carbohydrate value was detected during autumn (26.4 mg/100 g wet wt.). while the lowest value of 3.97 mg/100 gm wet wt. was detected in H1 during winter. The high salinity of H1 and low-temperature levels can decrease the carbohydrate contents which act synergistically to lower the metabolism activities of the microorganisms (Parvaiz & Satyawati, 2008). A strong negative correlation was observed between carbohydrates with TDS and major dissolved ions (*r* =  − 0.69 at *p* > 0.05) (Table 4). *ß*-carotene and phycocyanin showed their maximum content at H1 during winter (3.7 and 6.38 mg/100 g wet wt.) (Fig. 3). The elevation of ß-carotene and phycocyanin pigments in H1 resulted from flourishing of *A. salina* more than in H2. Moderate positive significant correlations were detected between ß-carotene, phycocyanin with orthophosphates, total phosphorus, and reactive silicate (*r* = 0.57, 0.51, and 0.60 for ß-carotene and 0.73, 0.81, and 0.64 for phycocyanin (Table 4).

The analysis of variance (ANOVA) showed a non-significant difference in the total protein levels among stations (*p* > 0.05), but there is a high significant difference among seasons (*p* = 0.002), while total carbohydrates and lipids have non-significant differences within different seasons (*p* > 0.05). At the same time, chlorophyll a, ß-carotene, and phycocyanin showed non-significant temporal differences.

### Biochemical contents in sediment, cyanobacterial mats, and *A. salina*

**Fig. 4:**
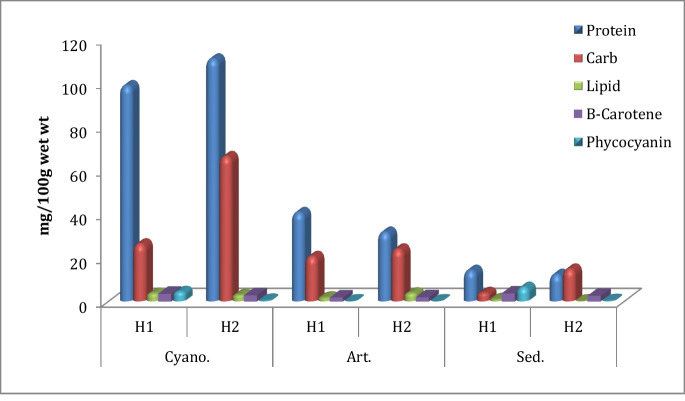
Comparison of biochemical contents of cyanobacteria, *A. salina*, and sediment in El-Hamra Lakes (mg/100 g wet wt.)

Cyanobacterial mats showed high levels of most biochemical components—i.e., total protein, carbohydrate, and lipid contents in H2 with values of 110.93, 66.25, and 3.68 mg/100 g wet wt., respectively. Cyanobacterial mats flourished in H2 more than in H1 due to their low salinity. Their values lowered in *A. salina* organisms in H1 reaching 40.58, 23.91, and 3.8 mg/100 mg wet wt. (Fig. 4). On the other hand, *ß*-carotene and phycocyanin in the sediment of H1 harbored the highest values among different compartments at 3.7 and 6.38 mg/100 g wet wt., respectively, and then followed by cyanobacteria in H1 at 3.51 and 4.5 mg/100 g wet wt., respectively. The considerable biochemical contents detected in sediment can be primarily attributed to the obvious thin pink microbial biofilm that appeared during the sample collection, the de-attached fragments of cyanobacterial mats, or the visibly disseminated *A. salina* cysts that contain a high amount of lipid, protein, and carbohydrates (Berdimbetova et al., 2019; Hasegawa et al., 2010; Navarro et al., 1992).

### Total phenolic contents

The total phenolic compounds (TPC), which originated from different microorganisms, have an essential role in investigating and understanding the biochemical and physiological behavior of these microorganisms (Jerez-Martel et al., 2017). Polyphenols and phenolic compounds are secondary metabolites with high antioxidant characteristics. The quantification of TPC gives important insights to understand the bacterial defense mechanisms against oxidative stress. TPC has significant implications for environmental, pharmaceutical, and agricultural sciences (Arguelles, 2022).

**Fig. 5:**
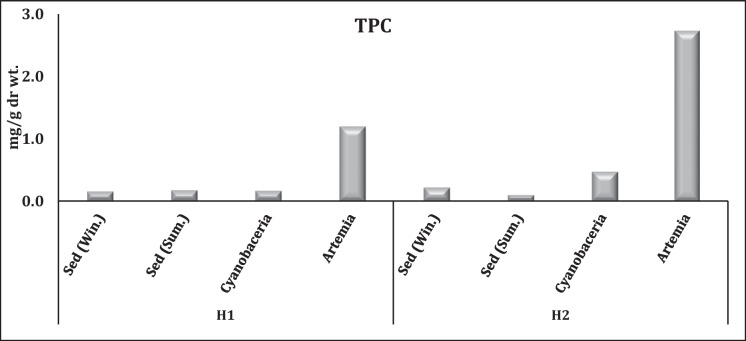
Variation of TPC contents in El-Hamra Lakes

TPC contents in sediment samples exhibited a non-significant variation—whether observed temporally or spatially—which indicates a consistent and stable presence over time and across different locations with annual averages of 0.165 and 0.161 mg/g dr. wt. for H1 and H2, respectively (Fig. 5). TPC levels in cyanobacterial films in H2 showed a threefold increase of 0.47 mg/g dr. wt., compared to 0.16 mg/g dr. wt. in H1. At the same time, TPC levels in *A. salina* in H2 (2.7 mg/g dr. wt.) were twofold higher than in H1 (1.19 mg/g dr. wt.).

### Total flavonoid contents

**Fig. 6:**
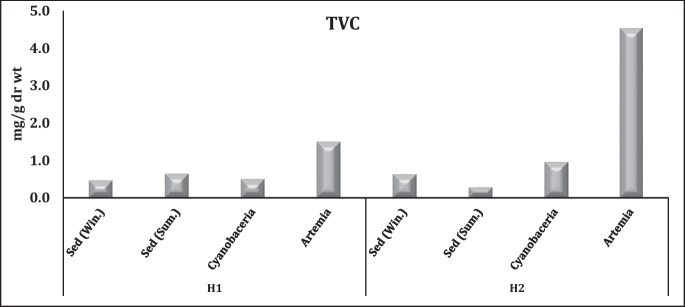
Total flavonoid compounds (TVC) contents in El-Hamra Lakes (mg Qu/g)

The total flavonoid compounds (TVC) are considered a subgroup of polyphenolic compounds; they have a broad spectrum of biological activities—particularly antimicrobial, anti-inflammatory, and antioxidant properties. In addition, TVCs have high medical and environmental importance as they provide promising antimicrobial effects which can contribute to the development of novel antibiotics (Shraim et al., 2021). TVC contents showed slight narrow variations in sediment samples with annual averages of 0.0.55 and 0.0.45 mg/g dr. wt. for H1 and H2, respectively (Fig. 6). On the other hand, TVC levels in cyanobacterial films in H2 showed ≈ a twofold increase (0.95 mg/g dr. wt.) compared to H1 (0.49 mg/g dr. wt.). At the same time, their levels of *A*. *salina* in H2 (4.5 mg/g dr. wt.) threefold exceeded the values in H1 (1.49 mg/g dr. wt.) (Fig. 6).

### Free radical scavenging activity by DPPH

**Fig. 7:**
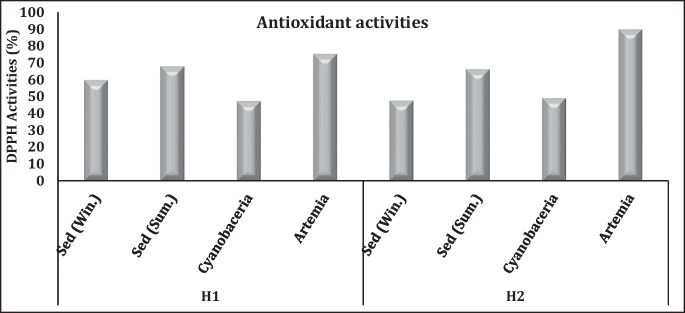
Free radical scavenging activity by DPPH in El-Hamra Lakes

The efficiency of antioxidants for certain bio-extracts was estimated with a common free radical scavenging activity method by using the DPPH (2,2-diphenyl-1-picrylhydrazyl) (Brand-William et al., 1995). The DPPH scavenging activities of methanolic crude extract from cyanobacterial films in El-Hamra Lake showed extremely narrow variation in the annual average of DPPH activities (47.73%) in H1 compared with 49.15% in H2 (Fig. 7). Surface sediment in H1 and H2 had higher free radical scavenging activities during summer than winter. On the other hand, the DPPH scavenging activities of *A. salina* organisms in H2 (*avr.* 89%) were higher than in H1 (*avr.* 75.2%), while the DPPH scavenging activities of crude extracts from different compartments of El-Hamra Lakes followed the order of DPPH (*A. salina*) > DPPH (sediment) > DPPH (cyanobacteria) (Fig. 7).

### In vitro, antimicrobial assays

The antimicrobial activities of sediment, cyanobacterial mats, and *A. salina* from Wadi El-Natrun Soda Lakes in polar and non-polar organic solvents were assayed against four bacterial and one yeast strains (Table 5). In general, the five microbes responded differently against different extracts. The growth of *P. aeruginosa* showed the least inhibition at different extracts from different sources. In general, the extermination of *P. aeruginosa* is extremely difficult due to its high resistance against antibiotics (Panga et al., 2019), cyanobacterial extracts (Khairy and El-Kassas, 2010; Abd El-Karim, 2016), and plant extracts (Barreca et al., 2014). The gram-positive bacterial strains, *S. aureus* and *B*. *cereus*, and the fungal *C. albicans* were highly susceptible to different extracts. Heidari et al. (2012) found that all polar and non-polar extracts of extremophilic cyanobacteria species can inhibit the growth of *S. aureus* and *C. albicans*. The extracts of *Lyngbya aestuarii* exhibited moderate to good antibacterial activity against all selected gram-positive reference strains (i.e., *S. aureus* and *B. subtilis)* (Hassouani et al., 2017). Different extracts of cyanobacterial mats from hypersaline lakes and hot springs were highly effective against the tested organisms: *S. aureus*, *B*. *cereus*, and the fungal *C. albicans* (Abd El-Karim, 2016; Attwa, 2021).

**Table 5:** Antimicrobial activities around the wells (inhibition zone in diameter, mm) of different organic extracts (50 µml/well) of sediments, cyanobacterial mats, and *A. salina* from Wadi El-Natrun Soda Lakes at a concentration of 5 mg/ml

	Pathogens	Sediment					Cyanobacterial mats					*A. salina*				
		EA	M	A	Ch	NH	EA	M	A	Ch	NH	EA	M	A	Ch	NH
*-ve*	*S. typhi*	8	10	-	8	7	9	15		14	12	9	15	8	13	15
	*P. aeruginosa*	-	-	9	-	-	-	10	7	-	-	-	11	-	-	13
+ *ve*	*S. aureus*	12	12	-	11	8	14	13	11	10	13	11	14	12	12	13
	*B*. *cereus*	9	-	7	13	9	10	19	8	16	19	14	22	16	17	17
Fungi	*C. albicans*	7	11	8	10	15	12	20	17	15	14	13	21	15	12	16

EA ethyl acetate, *M* methanol, *A* acetone, *Ch* chloroform, *NH* n-hexane

Different extracts of the three used materials: sediment, cyanobacterial mats, and *A. salina*, effectively inhibited different pathogens, which can be due to their considerable contents of total phenolic and total flavonoids. Moreover, fatty acid extracts of *A. salina* had high antimicrobial effects against *Vibrio anguillarum* and *Streptococcus agalactiae* (Amarouayache, 2017). The n-3 HUFA, DHA, EPA, α-linolenic, and caprylic acid were observed with high amounts in *A. salina* (Amarouayache et al., 2017). These fatty acids were also detected in cyanobacterial mats from hypersaline lakes (Mahmoud and Abd El-Karim, 2016).

The results of different extracts from sediment, cyanobacterial mats, and *A. salina* indicated that methanolic extracts were the most effective against different pathogenic strains. Between methanol, n-hexane, and ethyl acetate solvents, methanolic extracts showed the highest inhibition zones against *S. aureus*, *B. subtilis*, *P. aeuroginosa*, *Escherichia coli*. and *Klebsiella pneumoniae* (Gheda & Ismail, 2020). Moreover, good antibacterial activities of methanolic extracts have already been reported on four gram-positive and six gram-negative bacteria, and their reported zones of inhibition were 12 − 14 mm in diameter at higher doses (Abed et al., 2011). Between hexane, dichloromethane, chloroform, ethyl acetate, acetone, and methanol, the quantitative analysis showed that *S. platensis* methanolic extract exhibited higher inhibition against *P. aeruginosa* (LewisOscar et al., 2018).

## Conclusion

The comprehensive study of chemical characteristics of water of the soda lakes: El-Hamra 1 (H1) and El-Hamra 2 (H2) in Wadi El-Natrun, Egypt, considering their biochemical parameters, total phenolic, and flavonoid content, as well as antioxidant activities—provides valuable insights into the unique ecological dynamics of these extreme environments. The calculation of the equivalent percentage of dissolved major ions showed that the dominance sequence of major anions and cations followed the order of Cl^−^  > SO_4_^2−^  > CO_3_^−2^ > HCO_3_^−^ and Na^+^ > Mg^+2^ > K^+^  > Ca^+2^. These results help to identify and classify the chemical type of water in El-Hamra lakes precisely as a *soda-saline* chemical type. As revealed in the results, the pH of water exceeded 9.3 and Na^+^ ranked the first among major cations abundance (58%), and the summation of HCO_3_^−^  + CO_3_^2−^ (≈6%) was less than an abundance of Cl^−^ (≈ 71%) and SO_4_^−2^ (≈ 22%).

The recorded biochemical components, TPC and TVC, and antioxidant activities give essential highlights about the great adaptability of organisms, particularly cyanobacteria and brine shrimp (*A. salina*) in the studied soda lakes, giving insights into their resilience to extreme conditions. The total phenolic and flavonoid are present in concern contents in sediment, cyanobacteria, and brine shrimp (*A. salina*), which contributes to and enhances antioxidant capacity reaching 89%. The three used materials, sediment, cyanobacterial mats, and *A. salina,* effectively inhibit different pathogens.

## Data Availability

No datasets were generated or analysed during the current study.
